# Safety and clinical outcomes of rituximab therapy in patients with different autoimmune diseases: experience from a national registry (GRAID)

**DOI:** 10.1186/ar3337

**Published:** 2011-05-13

**Authors:** Hans-Peter Tony, Gerd Burmester, Hendrik Schulze-Koops, Mathias Grunke, Joerg Henes, Ina Kötter, Judith Haas, Leonore Unger, Svjetlana Lovric, Marion Haubitz, Rebecca Fischer-Betz, Gamal Chehab, Andrea Rubbert-Roth, Christof Specker, Jutta Weinerth, Julia Holle, Ulf Müller-Ladner, Ramona König, Christoph Fiehn, Philip Burgwinkel, Klemens Budde, Helmut Sörensen, Michael Meurer, Martin Aringer, Bernd Kieseier, Cornelia Erfurt-Berge, Michael Sticherling, Roland Veelken, Ulf Ziemann, Frank Strutz, Praxis von Wussow, Florian MP Meier, Nico Hunzelmann, Enno Schmidt, Raoul Bergner, Andreas Schwarting, Rüdiger Eming, Michael Hertl, Rudolf Stadler, Michael Schwarz-Eywill, Siegfried Wassenberg, Martin Fleck, Claudia Metzler, Uwe Zettl, Jens Westphal, Stefan Heitmann, Anna L Herzog, Heinz Wiendl, Waltraud Jakob, Elvira Schmidt, Klaus Freivogel, Thomas Dörner

**Affiliations:** 1 Medizinische Klinik und Poliklinik II, Universitätsklinikum Würzburg, Klinikstr 6-8, 97070 Würzburg, Germany; 2 Department Medicine/Rheumatology and Clinical Immunology, Charité-Universitätsmedizin Berlin, Schumannstr 20/21, 10098 Berlin, Germany; 3 Medizinische Poliklinik, Klinikum der Universität München, Pettenkoferstr. 8a, 80336 München, Germany; 4 Department of Internal Medicine II, Universitätsklinikum Tübingen, Otfried-Müller-Str. 10, 72 076 Tübingen, Germany; 5 Department of Neurology, Jüdisches Krankenhaus Berlin, Heinz-Galinski-Strasse 1, 13347 Berlin, Germany; 6 Internal Medicine Rheumatology, Krankenhaus Dresden-Friedrichstadt, Friedrich Strasse 41, 01067 Dresden, Germany; 7 Internal Medicine ICU, Medizinische Hochschule Hannover, Carl-Neuberg-Str. 1, 30625 Hannover, Germany; 8 Endokrinologie, Diabetologie und Rheumatologie, Heinrich Heine-Universität Düsseldorf, Moorenstr. 5, 40225 Düsseldorf, Germany; 9 Klinik für Innere Medizin I, Uniklinik Köln, Josef-Stelzmann-Str 9, 50931 Köln, Germany; 10 Department of Rheumatology and Clinical Immunology, Kliniken Essen-Süd, Propsteistr. 2, 45239 Essen, Germany; 11 Klinikum Augsburg, Stenglinstr., 86156 Augsburg, Germany; 12 University Hospital Schleswig-Holstein Campus Lübeck, Universität Lübeck/Klinikum Bad Bramstedt, Oskar-Alexander-Straße 26, 24576 Bad Bramstedt, Germany; 13 Department of Internal Medicine and Rheumatology, Universität Giessen/Kerckhoff-Klinik, 61231 Bad Nauheim, Germany; 14 ACURA Rheumazentrum Baden-Baden, Red River Valley Road 5, 76530 Baden-Baden, Germany; 15 Ambulantes Rheumazentrum, Argentinische Allee 42, 14163 Berlin, Germany; 16 Department of Dermatology, Technische Universität Dresden, Haus 105 auf der Blasewitzer Str. 86, 01304 Dresden, Germany; 17 Department of Dermatology, Universitätsklinikum Erlangen, Hartmannstrasse 14, 91054 Erlangen, Germany; 18 Klinik für Neurologie, Klinikum der Johann Wolfgang Goethe-Universität, Theodor-Stern-Kai 7, 60590 Frankfurt, Germany; 19 Zentrum Innere Medizin Abt. Nephrologie/Rheumatologie, Universitätsmedizin Göttingen, Robert-Koch-Str. 40, 37075 Göttingen, Germany; 20 Praxis für Hämatologie und Internistische Onkologie, Rösebeckstr. 15, 30449 Hannover, Germany; 21 Rheumapraxis Hofheim, Reifenberger Strasse 6, 65719 Hofheim, Germany; 22 Klinik und Poliklinik für Dermatologie und Venerologie, Universität zu Köln, Kerpener Straße 62, 50937 Köln, Germany; 23 Klinik für Dermatologie, Allergologie und Venerologie, Universität, Ratzeburger Allee 160, 23538 Lübeck, Germany; 24 Medizinische Klinik A, Klinikum der Stadt Ludwigshafen, Bremserstr. 79, 67063 Ludwigshafen, Germany; 25 Medizinische Klinik, Universitätsmedizin der Johannes Gutenberg-Universität Mainz, Langenbeckstr. 1, 55131 Mainz, Germany; 26 Abteilung von Dermatologien und Allergology, Philipps Universität Marburg, Deutschhausstrasse 9, 35033 Marburg, Germany; 27 Department of Dermatology, Johannes Wesling Klinikum Minden, Hans-Nolte-Straße 1, 32429 Minden, Germany; 28 Facharzt f. Innere Medizin-Rheumatologie, Evangelisches Krankenhaus, Marienstr. 11, 26121 Oldenburg, Germany; 29 Rheumatologe, Ev. Fachkrankenhaus Ratingen, Rosenstr. 2, 40882 Ratingen, Germany; 30 Rheumatologie, Universitätsklinikum Regensburg, Franz-Josef-Strauß-Allee 11, 93053 Regensburg, Germany; 31 Medizinische Klinik II, Krankenhaus der Barmherzigen Brüder, Prüfeninger Str. 86, 93049 Regensburg, Germany; 32 Klinik für Neurologie und Poliklinik, Universitätsklinikum Rostock, Gehlsheimer Straße 20, 18147 Rostock, Germany; 33 Praxis für Allgemeinmedizin, Goethestr. 35, 78669 Schramberg-Sulgen, Germany; 34 Schwerpunkt Rheumatologie und klinische Immunologie, Marienhospital Stuttgart, Böheimstr. 37, 70199 Stuttgart, Germany; 35 Abteilung der Neurologie, Universität Würzburg, Josef-Schneider-Str. 11, 97080 Würzburg, Germany; 36 Analytica International GmbH, Untere Herrenstr. 25, 79539 Lörrach, Germany

## Abstract

**Introduction:**

Evidence from a number of open-label, uncontrolled studies has suggested that rituximab may benefit patients with autoimmune diseases who are refractory to standard-of-care. The objective of this study was to evaluate the safety and clinical outcomes of rituximab in several standard-of-care-refractory autoimmune diseases (within rheumatology, nephrology, dermatology and neurology) other than rheumatoid arthritis or non-Hodgkin's lymphoma in a real-life clinical setting.

**Methods:**

Patients who received rituximab having shown an inadequate response to standard-of-care had their safety and clinical outcomes data retrospectively analysed as part of the German Registry of Autoimmune Diseases. The main outcome measures were safety and clinical response, as judged at the discretion of the investigators.

**Results:**

A total of 370 patients (299 patient-years) with various autoimmune diseases (23.0% with systemic lupus erythematosus, 15.7% antineutrophil cytoplasmic antibody-associated granulomatous vasculitides, 15.1% multiple sclerosis and 10.0% pemphigus) from 42 centres received a mean dose of 2,440 mg of rituximab over a median (range) of 194 (180 to 1,407) days. The overall rate of serious infections was 5.3 per 100 patient-years during rituximab therapy. Opportunistic infections were infrequent across the whole study population, and mostly occurred in patients with systemic lupus erythematosus. There were 11 deaths (3.0% of patients) after rituximab treatment (mean 11.6 months after first infusion, range 0.8 to 31.3 months), with most of the deaths caused by infections. Overall (*n *= 293), 13.3% of patients showed no response, 45.1% showed a partial response and 41.6% showed a complete response. Responses were also reflected by reduced use of glucocorticoids and various immunosuppressives during rituximab therapy and follow-up compared with before rituximab. Rituximab generally had a positive effect on patient well-being (physician's visual analogue scale; mean improvement from baseline of 12.1 mm).

**Conclusions:**

Data from this registry indicate that rituximab is a commonly employed, well-tolerated therapy with potential beneficial effects in standard of care-refractory autoimmune diseases, and support the results from other open-label, uncontrolled studies.

## Introduction

Research into the pathogenesis of autoimmune diseases has led to a greater understanding of the function of the immune cells, and in particular to the role of B cells in innate and adaptive immunity [1-4]. B cells act as antigen-presenting cells, are precursors of autoantibody-producing cells, and produce proinflammatory and anti-inflammatory cytokines and chemokines that assist the activation of T cells, all of which may contribute to the pathogenesis of autoimmune diseases [1,5]. Consequently, interest in B cells as a target in the treatment of autoimmune disease continues to grow [6].

Preliminary data indicate that B cell depletion may be effective in autoimmune disease in the areas of rheumatology, nephrology, neurology and dermatology [7]. A greater amount of evidence for the effectiveness of B cell depletion has been gathered in rheumatoid arthritis (RA), with recent emerging data indicating that B cell depletion may also be effective in the treatment of antineutrophil cytoplasmic antibody (ANCA)-associated granulomatous vasculitis [8-10]. Rituximab, a monoclonal antibody that selectively targets CD20+ B cells and leads to their depletion, has demonstrated significant efficacy and a good safety profile in clinical trials conducted in patients with active RA [11-17]. The long-term efficacy and safety of rituximab in RA is particularly relevant as many of the autoimmune diseases are relatively rare, and as such, clinical development of drugs for these conditions will be less likely.

The currently available evidence provides a confusing picture as to the benefit:risk profile of rituximab in various autoimmune diseases, although the bulk of evidence comes from small studies of off-label use [18-55]. There is also a discrepancy between placebo-controlled clinical trials [56-59] and real-life registry data [60,61], where patients receiving rituximab mostly had standard of care (SOC)-refractory disease [62]. Therefore, the German Registry of Autoimmune Diseases (GRAID) was established to provide further evidence on the safety and clinical outcomes of rituximab in patients with autoimmune diseases who were enrolled across rheumatology, dermatology, neurology, and nephrology, and were mostly SOC-treatment refractory.

## Materials and methods

### Study design

GRAID was a multicentre, non-interventional, retrospective study of patients with autoimmune diseases. Patients who were included in the study had received a regimen of rituximab that was deemed appropriate by their treating physician. As patients received rituximab off-label, the regimens of patients included in the registry varied across the different autoimmune diseases.

A total of 42 German centres were involved, including university and other large hospitals as well as private practices, and included physicians from rheumatology, haematology, nephrology, neurology, dermatology and internal medicine specialities. Prior to patient selection, participating physicians were informed of the inclusion and exclusion criteria, and received guidance on data collection. In brief, physicians who had agreed to participate were asked to provide data retrospectively on any patient who had a diagnosis of an autoimmune disease, excluding RA and non-Hodgkin's lymphoma (NHL), who had received rituximab up to and including 31 August 2008. Data were collected retrospectively by the principal investigators of the centres using a web-based electronic case report form (eCRF). The eCRF was designed to only permit entry of the target population, and was specifically programmed to exclude RA and NHL patients, and any inconsistencies in data collection. Data for a selection of 14 diagnoses could be entered. In the case of non-pre-defined diseases, these were classified as 'other autoimmune diseases' (Table 1). The registry was open for data entry between 20 December 2008 and 31 July 2009.

**Table 1:** Patient demographics and mean doses of rituximab received by patients with various autoimmune diseases

Diagnosis	Patients, n (%)	Total observation time, patient-years	Mean (SD) rituximab dose, mg
***Total***	***370***	***299***	***2,440 (1,295)***
Arthritis (non-RA)/ankylosing spondylitis/psoriatic arthritis	6 (1.6)	3.8	2,333 (816)
Autoimmune haemolytic anaemia	3 (0.8)	1.6	2,233 (204)
Autoimmune thrombocytopenia	10 (2.7)	6.5	2,602 (1,047)
Glomerulonephritis	9 (2.4)	7.0	2,150 (1,660)
Cryoglobulinaemic vasculitis	5 (1.4)	4.1	2,576 (971)
Wegener's granulomatosis/microscopic polyangiitis	58 (15.7)	61.4	3,008 (1,524)
Multiple sclerosis/neuromyelitis optica	56 (15.1)	48.3	2,679 (1,252)
Myasthenia gravis	5 (1.4)	2.7	1,890 (1,107)
Pemphigus	37 (10.0)	22.7	1,755 (1,163)
Sjögren's syndrome	6 (1.6)	4.3	2,271 (995)
Polydermatomyositis	26 (7.0)	23.4	2,634 (1,810)
Systemic lupus erythematosus	85 (23.0)	66.8	2,331 (1,033)
Vasculitis	13 (3.5)	9.5	2,277 (1,168)
Overlap syndromes: mixed connective tissue disease	19 (5.1)	16.3	2,550 (1,031)
Others^a^	32 (8.6)	20.8	2,079 (1,224)

RA, rheumatoid arthritis; SD, standard deviation.

^a^Other autoimmune diseases included: Felty syndrome (*n *= 1); antiphospholipid syndrome (*n *= 1); chronic inflammatory demyelinating polyneuropathy (CIDP) (*n *= 3); anti-myelin associated glycoprotein associated with CIDP (*n *= 1); anti-Scl70-positive systemic sclerosis with myositis (*n *= 1); thrombotic thrombocytopenic purpura (*n *= 2); epidermolysis bullosa acquisita (*n *= 3); bullous pemphigoid (*n *= 1); mucous membrane pemphigoid (*n *= 1); uveitis (*n *= 1); Behçet disease (*n *= 1); systemic sclerosis (*n *= 1); systemic sclerosis with CREST syndrome (*n *= 1); diffuse cutaneous sclerosis (*n *= 1); common variable immunodeficiency (*n *= 1); Castleman's disease (*n *= 1); human immunodeficiency virus and myopathy (*n *= 1); Still's disease (*n *= 1); Crohn's disease (*n *= 1); Lambert-Eaton myasthenic syndrome (*n *= 1); autoimmune neuropathy (*n *= 1); stiff-person syndrome (*n *= 1); unspecified vasculitis (*n *= 1); anti-Hu positive encephalopathy of the brain stem (*n *= 1); acquired factor VIII haemophilia (*n *= 1); sarcoidosis (*n *= 1); diagnosis not specified (*n *= 1).

Ethical approval and approval by the local data protection agency were obtained by Charite Universitätsmedizin Berlin. Where required, because of local regulations, local committee approval was obtained by the principal investigators.

### Patients

Patients enrolled on the register were aged ≥ 18 years with an autoimmune diagnosis other than RA or NHL and an inadequate response to previous SOC. There was a requirement that rituximab must have started on or before 31 August 2008, with the last follow-up before 20 December 2008. There were no restrictions placed on time to follow-up, although there was a cut-off for data entry of 31 July 2009. Although informed consent was not obtained from the participating patients because of the retrospective nature of this analysis, patients' data were protected as follows: all patients' data were entered into the register under a pseudonym according to current standards, which meant that only the physicians of each centre entering data via the eCRF had access to the code for participating patients.

### Assessments

Clinically adverse events (AEs), including infections, were recorded throughout the treatment and follow-up period of the study. The eCRF included supplementary information on the standardised grading system for the recording of AEs. The intensity of AEs was graded using the National Cancer Institute (NCI) Common Terminology Criteria for Adverse Events (CTCAE), version 3 or a Grade 1-5 severity scale. Similarly, serious AEs (SAEs) were defined as per the International Conference on Harmonization (ICH) criteria. Infusion-related reactions (IRRs) were identified by a glossary of MedDRA terms for AEs occurring during or within 24 hours of an infusion.

Different assessment tools are used to measure changes in disease activity for the different autoimmune diseases; therefore, to enable an approximate comparison across the diseases, clinical response was categorised as complete response, partial response and no response, as judged at the discretion of the investigators. The use of certain co-therapies prior to, during and after rituximab therapy, and patients' well-being were also assessed. Patient well-being was estimated using a physician's visual analogue scale (VAS), which was measured on a scale of 0 to 100 mm, with 100 mm indicating maximum well-being.

### Statistical analysis

All patients who were entered into the register were included in the safety analysis, whereas only patients with at least one control visit were included in the clinical outcomes analysis. Analysis with standard descriptive statistics (mean, median) was performed, as well as analysis of variance (ANOVA) testing for differences between centres and disease types. Poisson regression was used to investigate the relationship between infection rates and disease state. To determine any differences in distribution patterns of response across the different diseases, responses of patients for individual diseases were analysed using the chi-squared test and a row mean difference test.

## Results

### Patient disposition and treatment

A total of 370 patients with a diagnosis of an autoimmune condition other than RA or NHL and treated with rituximab provided a total of 299 patient-years of observation in this study population (Table 1; further detail of the follow-up period for each diagnosis is provided in the Additional file 1). The most common diagnosis was SLE (23.0% of patients), with the least represented disorder being autoimmune haemolytic anaemia (0.8% of patients).

A total of 86% of patients received > 1 infusion with rituximab. Of the total rituximab infusions received across all diagnoses, the majority of patients received two (39.2% of patients) or four (34.1%) infusions, followed by those who received only one infusion of rituximab (13.5%); three (0.8%) patients received > 8 infusions of rituximab (further detail of the number of rituximab infusions is provided in Additional file 1). Most patients (77.6%) received one course of rituximab with 18.6% of patients having received two courses. The mean dose of rituximab received by the patients was 2,440 mg/patient over a median period of 194 (180 to 1,407) days. Patients with ANCA-associated granulomatous vasculitis received the highest doses of rituximab (mean dose/patient 3,008 mg) and most conditions were treated with > 2,000 mg of rituximab per patient during this study (Table 1). Only patients with myasthenia gravis or pemphigus received less than 2,000 mg over the study period.

### Safety

**Figure 1:**
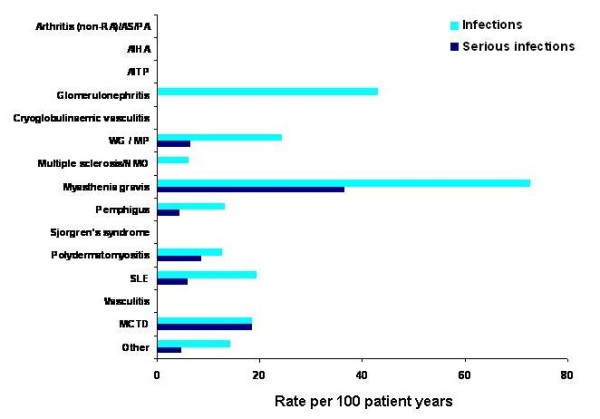
**Rates of infection and serious infections in patients with various autoimmune diseases who received rituximab**. The frequencies depicted are based on different sizes of the patient groups (see Table 1). ANOVA testing for heterogeneity between the patient groups did not provide significant differences between the patient groups. AIHA, autoimmune haemolytic anaemia; AITP, autoimmune thrombocytopenia; AS, ankylosing spondylitis; MCTD, mixed connective tissue disease; MP, microscopic polyangiitis; NMO, neuromyelitis optica; PA, psoriatic arthritis; RA, rheumatoid arthritis; SLE, systemic lupus erythematosus; WG, ANCA-associated granulomatous vasculitis.

The majority of patients (87.8%) had no documented infection. During treatment with rituximab, there was an overall rate of infection of 18.1 per 100 patient-years. The patient groups with the highest rate of infection were those with glomerulonephritis (43 per 100 patient-years) and myasthenia gravis (72.8 per 100 patient-years) (Figure 1). However, these patient groups were rather small and, therefore, statistical analysis (ANOVA) did not identify a disease-related higher risk for infections.

**Figure 2:**
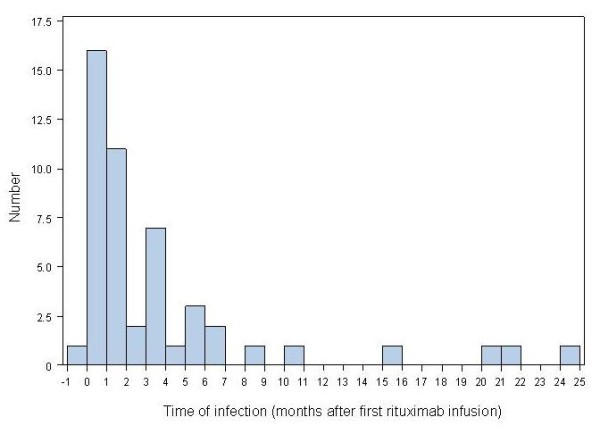
**Distribution of total number of infections over time following rituximab infusion in patients with autoimmune diseases**.

The majority of infections were mild (3.8% of the overall population) or moderate (3.2%) in severity, although 3.7% of patients had severe infections. An analysis of the distribution of infections showed that the majority of clinically relevant infections occurred within seven months of the first rituximab infusion (Figure 2). The majority of the infections were bacterial (*n *= 26), with the remainder being of viral (*n *= 9), fungal (*n *= 4) or unknown (*n *= 5) origin. Interestingly, only very few patients had more than one infection during the study: there were two different infections in three patients and three different infections in two patients. Thus, in most cases only one infection was recorded during the observation period of the study.

For serious infections, the overall rate was 5.3 per 100 patient-years during rituximab therapy. Rates of serious infections were generally low across all the conditions studied (Figure 1). The greatest rate of serious infections was reported by patients with myasthenia gravis (36.4 per 100 patient-years). The high rate of infections and serious infections in patients with myasthenia gravis was a result of infections occurring within a relatively short observation period (2.7 patient-years). However, there was no statistically (ANOVA) significant relationship between infection rates and any diagnosis; although, there was a significant relationship between higher doses of rituximab and a reduced rate of infections (Poisson regression model).

Opportunistic infections were infrequent across the whole study population, and mostly occurred in patients with SLE. There were five opportunistic infections recorded in patients with SLE. Bacterial infections, considered and recorded by the individual participating physicians as opportunistic infections, included a gastrointestinal infection caused by *Salmonella typhimurium *(*n *= 1), meningococcal meningitis (*n *= 1) and *Listeria meningitis *(*n *= 1). The remaining two patients with SLE had outbreaks of oesophageal candidiasis and Herpes zoster infection. There were also two other cases of Herpes zoster reactivation, one each in patients with Wegener's granulomatosis and pemphigus.

**Figure 3:**
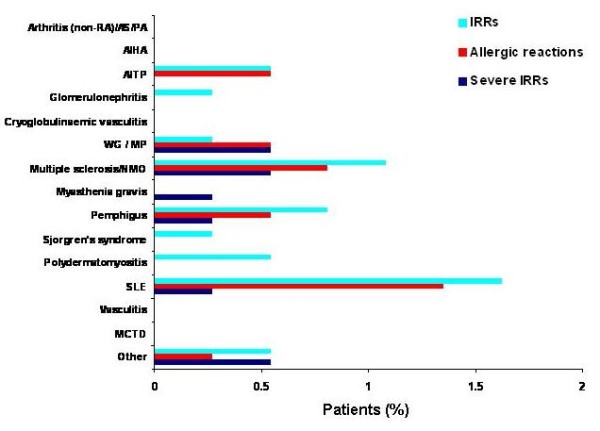
**Proportion of patients with various autoimmune diseases who had infusion-related reactions (IRRs), allergic reactions and withdrawals because of severe IRRs during rituximab therapy**. AIHA, autoimmune haemolytic anaemia; AITP, autoimmune thrombocytopenia; AS, ankylosing spondylitis; MCTD, mixed connective tissue disease; MP, microscopic polyangiitis; NMO, neuromyelitis optica; PA, psoriatic arthritis; RA, rheumatoid arthritis; SLE, systemic lupus erythematosus; WG, ANCA-associated granulomatous vasculitis.

During treatment with rituximab, overall, there were 22 (5.9%) IRRs, 15 (4.1%) allergic reactions, 9 (2.4%) severe IRRs leading to discontinuation of therapy and 21 (5.7%) undifferentiated IRRs. When IRRs, allergic reactions and severe IRRs were distributed by autoimmune condition, no more than 2% of patients were affected, with no significantly enhanced rate or type of reaction in any particular disease (*P *= 0.65, chi-squared test) (Figure 3).

Of the 370 patients, there have been 11 deaths (3.0% of patients) reported following treatment with rituximab (Table 2). Patients with ANCA-associated granulomatous vasculitis were the group most affected (four deaths), followed by those with polydermatomyositis (three deaths). Infection was the major cause of death, which was the cause in seven cases. The timing of the 11 deaths after rituximab therapy (mean 11.6 months) ranged between 0.8 and 31.3 months after the first infusion of rituximab. Notably, the study protocol required documentation within six months after the last rituximab therapy. Thus, five deaths occurred in this period; for the remaining six deaths, these occurred outside the protocol-specified period of six months and, therefore, the data of this patient group are less certain. Patients (*n *= 7) with a short interval between rituximab infusion and death (< 7 months) had highly active, uncontrolled disease and received rituximab as a final option during intensive care therapy. Four of the deaths occurred more than 11 months after rituximab therapy. Two patients with ANCA-associated granulomatous vasculitis died 13.8 and 11.1 months after the last infusion of rituximab (31.3 and 12.7 months, respectively, after the first infusion) and two patients with polydermatomyositis died at 12.7 and 14.2 months following first and only infusion. Notably, within the largest patient groups of SLE and MS, no deaths were reported.

**Table 2:** Number and cause of deaths according to autoimmune disease diagnosis

Cause of death	Diagnosis	Total, n
Infection	Wegener's granulomatosis/microscopic polyangiitis (*n *= 2) Myasthenia gravis (*n *= 1) Polydermatomyositis (*n *= 2) Mixed connective tissue disease (*n *= 1) Other (*n *= 1)	7
Other, unspecified	Wegener's granulomatosis/microscopic polyangiitis (*n *= 1) Multiple sclerosis/neuromyelitis optica (*n *= 1) Polydermatomyositis (*n *= 1)	3
Infection plus other event	Wegener's granulomatosis/microscopic polyangiitis (*n *= 1)	1

In terms of co-therapy (Table 3), it needs to be emphasised that prior to rituximab therapy almost all patients received immunosuppressives (98.1%) together with a high frequency of glucocorticoids, intravenous immunoglobulin (Ig) (17.0%) and plasmapheresis (13.8%), consistent with a very refractory patient population. In addition, the frequency of usage of almost all of these therapies substantially decreased during therapy with rituximab, with the exception of prednisolone use which was comparable before and during rituximab (68.9% versus 66.1%). After rituximab therapy, the proportion of patients using other therapies remained lower than use prior to rituximab, although the use of immunosuppressives showed a trend towards greater use compared with during rituximab (Table 3).

**Table 3:** Overall comparison of the frequency of patients with autoimmune diseases

	Proportion (%) of patients receiving co-therapies		
	
	Before rituximab	During rituximab	After rituximab during follow-up^a^
Immunosuppressives	98.1	41.2	78.6
Prednisolone	68.9	66.1	58.9
with glucocorticoid bolus therapy	17.0	1.25	1.6
Methylprednisolone	25.4	13.2	4.3
Methotrexate	22.1	6.9	8.9
Cyclophosphamide	36.2	8.15	3.2
Azathioprine	38.1	11.9	10.8
Mycophenolate mofetil	27.6	15.0	17.8
Intravenous Ig	17.0	3.4	5.1
Plasmapheresis	13.8	3.4	0.8
Other immunosuppressives	16.0	5.3	5.4

Overall comparison of the frequency of patients with autoimmune diseases receiving at least one administration of different types of therapies before, during and after rituximab therapy. Note, that certain immunosuppressive drugs were substantially different between individual diseases and only frequencies above 10% have been considered for this comparison.

^a^Follow-up was as defined and recorded by the investigating physician.

### Clinical outcomes

**Figure 4:**
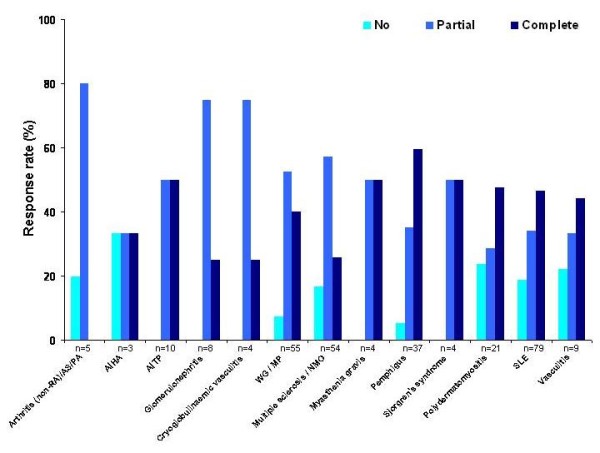
**Distribution of global response rates (full, partial versus no response) in patients with various autoimmune diseases who received rituximab, as reported by the treating physician over a median (range) of 194 (180 to 1,407) days according to the underlying diagnosis**. AIHA, autoimmune haemolytic anaemia; AITP, autoimmune thrombocytopenia; AS, ankylosing spondylitis; MP, microscopic polyangiitis; NMO, neuromyelitis optica; PA, psoriatic arthritis; RA, rheumatoid arthritis; SLE, systemic lupus erythematosus; WG, ANCA-associated granulomatous vasculitis.

Of the 370 patients, 293 patients had at least one control visit and, therefore, were included in the clinical outcomes analysis. In general, a low number of patients with autoimmune diseases had been classified as having no response to rituximab (Figure 4). In the overall study population independent of the underlying disease, 39 patients (13.3%) showed no response, 132 (45.1%) showed a partial response and 122 (41.6%) showed a complete response. There was a trend towards patients with no response having received a lower mean dose of rituximab compared with those with a partial or complete response (Table 4). When separated out into the various autoimmune diseases, all patients with Sjögren's syndrome, autoimmune thrombocytopenia, glomerulonephritis, cryoglobulinaemic vasculitis and myasthenia gravis were shown to have a response to rituximab (partial or complete) (Figure 4). However, these patient groups were small and bias can therefore not be excluded. When comparing the response rates between different patient groups, there was no significant difference using strict statistical methods (row mean scores differ, *P *= 0.26), including chi-squared test (*P *= 0.0872).

**Table 4:** Doses of rituximab stratified by response in patients with different autoimmune diseases

	Mean (SD) dose, mg		
	
Diagnosis	No response	Partial response	Complete response
***Total***	***n = 39*** ***1,990 (798)***	***n = 132*** ***2,683 (1,408)***	***n = 122*** ***2,528 (1,346)***
Arthritis (non-RA)/ankylosing spondylitis/psoriatic arthritis	*n *= 1 2,000 (-)	*n *= 4 2,500 (1,000)	-
Autoimmune haemolytic anaemia	*n *= 1 2,320 (-)	*n *= 1 2,000 (-)	*n *= 1 2,380 (-)
Autoimmune thrombocytopenia	-	*n *= 5 2,248 (1,321)	*n *= 5 2,956 (641)
Glomerulonephritis	-	*n *= 6 1,625 (826)	*n *= 2 4,100 (2,970)
Cryoglobulinaemic vasculitis	-	*n *= 3 2,960 (1,154)	*n *= 1 2,000 (-)
Wegener's granulomatosis/microscopic polyangiitis	*n *= 4 2,193 (561)	*n *= 29 3,474 (1,642)	*n *= 22 2,586 (1,384)
Multiple sclerosis/neuromyelitis optica	*n *= 9 2,022 (570)	*n *= 31 2,678 (903)	*n *= 14 3,157 (1,992)
Myasthenia gravis	-	*n *= 2 2,500 (707)	*n *= 2 1,225 (1,662)
Pemphigus	*n *= 2 1,500 (707)	*n *= 13 1,770 (899)	*n *= 22 1,769 (1,352)
Sjögren's syndrome	-	*n *= 2 2,312 (441)	*n *= 2 3,000 (1,414)
Polydermatomyositis	*n *= 5 1,700 (447)	*n *= 6 3,900 (3,209)	*n *= 10 2,818 (1,003)
Systemic lupus erythematosus	*n *= 15 2,121 (1,099)	*n *= 27 2,440 (1,119)	*n *= 37 2,499 (926)
Vasculitis	*n *= 2 1,500 (707)	*n *= 3 2,200 (346)	*n *= 4 3,000 (1,155)

RA, rheumatoid arthritis; SD, standard deviation.

**Figure 5:**
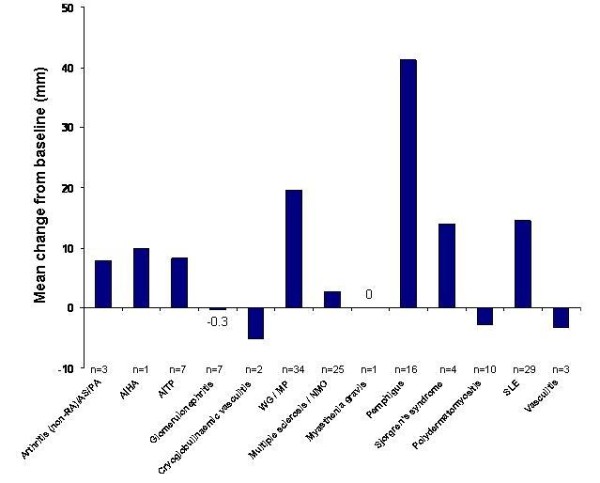
**Mean changes from baseline in patients' well-being over a median (range) of 194 (180 to 1,407) days during rituximab therapy in patients with various autoimmune diseases, as measured using the visual analogue scale (VAS; 0 to 100 mm) rated by the treating physician**. VAS scores were not assessed in patients with mixed connective tissue disease or 'other' autoimmune diseases. AIHA, autoimmune haemolytic anaemia; AITP, autoimmune thrombocytopenia; AS, ankylosing spondylitis; MP, microscopic polyangiitis; NMO, neuromyelitis optica; PA, psoriatic arthritis; RA, rheumatoid arthritis; SLE, systemic lupus erythematosus; WG, ANCA-associated granulomatous vasculitis.

Rituximab generally had a positive effect on patient well-being. Overall, there was an improvement from baseline in the estimation of patients' well-being (physician's VAS; mean improvement of 12.1 mm) following rituximab (Figure 5). Patients with pemphigus had the greatest improvement in well-being (from 33.8 to 75.0 mm). Patients with polydermatomyositis, vasculitis, glomerulonephritis and cryoglobulinaemic vasculitis showed slight reductions in VAS scores following rituximab, whereas patients with myasthenia gravis showed no change in VAS scores.

## Discussion

This study was designed to assess the safety and clinical outcomes of rituximab in refractory patients with various autoimmune diseases in a real-life clinical setting. Generally, rituximab was well tolerated across a number of autoimmune diseases and patients tended to have at least a partial response to therapy. Furthermore, rituximab was generally shown to have a positive effect on patient well-being, although it should be noted that this conclusion is based on a subjective outcome measure and so should be considered with caution.

In the current analysis across autoimmune diseases, the overall rate of infections and serious infections compares well with analyses of rituximab in RA [17] and SLE [63]. There are few studies that provide controlled trial data in other autoimmune diseases; however, in the HERMES study where relapsing-remitting multiple sclerosis was treated with rituximab or a placebo, a greater proportion of patients in the placebo group had serious infectious events than those receiving rituximab (5.7% versus 2.9%) [56]. The strict exclusion criteria employed in this study restricted entry of high-risk patients, and so this study does not truly reflect what might be expected in a real-life clinical setting. Overall, the current evidence would suggest that serious infections occur in a small proportion of patients with autoimmune diseases during rituximab therapy. In contrast, two controlled studies in SLE (EXPLORER) and lupus nephritis (LUNAR) have shown slightly elevated serious infection rates of 9.5% [59] and 16.4% [58], respectively, during rituximab therapy in combination with SOC (including mycophenolate mofetil (in EXPLORER and LUNAR), and methotrexate and azathioprine (in EXPLORER)) and high-dose corticosteroids, which might indicate that the regimens studied may not have been appropriate for these populations. An elevated serious infection rate was also reported in the rituximab group of the RITUXIVAS study, although, these patients with ANCA-associated vasculitis also received cyclophosphamide and high-dose corticosteroids as part of the regimen, and no differences in the rates were shown as compared with the control group, which received a cyclophosphamide-based regimen (18% for each regimen) [20]. In these three studies, serious infection rates were similar or elevated in the control groups compared with the rituximab regimens. Therefore, and in addition to, a potential relationship with underlying disease, concomitant immunomodulating therapies cannot be ruled out as a cause [20,58,59]. In particular, corticosteroids have an important additional effect on T cells and have been shown to be an important predisposing factor for infections [64]. Moreover, reduced IgG levels at baseline have been identified as risk factor for infections in RA [17] for which insufficient data were available in the registry to search for their role in modulating infectious risks and so further studies are needed. Data on Ig levels and B cell depletion were lacking from this current analysis, and therefore, these data are needed in subsequent registry studies.

Of interest is that the infection (serious and non-serious) rates over time remained stable with multiple courses of rituximab in RA [17]. In addition, it has been reported that retreatment with rituximab based on treatment to target DAS28 remission in patients with RA compared with an 'on demand' approach resulted in better disease control with comparable safety profiles [65]. Furthermore, retreatment with rituximab at a fixed interval of six months provided greater improvements in disease control at one year with a serious infection rate similar to that in patients with RA who received a single course [15,66]. These data might suggest that control over disease activity may be closely related to safety and the risk of infections. With the suggestion from the current clinical outcomes data that responses may be improved with greater doses of rituximab in patients with various autoimmune diseases, a stable safety profile over time will provide physicians with the confidence to prescribe multiple courses of rituximab for patients with autoimmune conditions. Although most of the complex systemic diseases reported here may require higher doses *per se*, dose-findings studies for rituximab within the individual diagnoses are still lacking and preclude any far-reaching conclusions.

A significant relationship between higher doses and a reduced rate of infections (Poisson regression model) was also demonstrated in this analysis. However, it cannot be excluded that infections led to discontinuation of therapy and resulted in lower treatment doses. Indeed, the majority of clinically relevant infections occurred within seven months of the first rituximab infusion, likely reflecting that severe infections led to discontinuation of rituximab. Therefore, a selection bias of patients initially developing infections remains possible. Alternatively, control of disease activity by successful rituximab therapy may have led to reduced disease activity and reduced subsequent infections as suggested by fixed-dosing intervals of rituximab in RA [65]. The overall safety data of this registry do not indicate that certain diseases may have a higher risk for any adverse event, including a higher propensity of infections. This conclusion can be further substantiated by the observation that during rituximab therapy the lowest frequencies of certain co-therapies were registered (Table 3), which reflects the overall clinical effect by this B cell directed therapy as well as that these co-therapies likely do not substantially confound the safety database. The increase of various therapeutic modalities after rituximab therapy is likely related to increased disease activity or when insufficient response to rituximab required new treatments.

Although a direct comparison cannot be made between the safety analysis of rituximab in patients with RA [17] and the current study for several reasons (heterogeneous patient populations, different dosing regimens and different study periods), it appears from the long-term analysis in RA that IRRs diminish over time; therefore, it would not be unreasonable to expect that the rate of IRRs over time in various autoimmune diseases might decrease with each course of rituximab. However, caution should be taken in interpreting these data as patients with more severe IRRs may discontinue rituximab and, therefore, provide a distorted impression. In addition, IRRs were defined in this analysis as events that occurred during or within 24 hours of an infusion, which does not capture those that may have occurred after one week, for example; therefore, the data recorded in this registry may represent an under-reporting of the level of IRRs. Fortunately, the vast experience with rituximab in RA and NHL has led to effective techniques in managing these infusion-related complications.

In the RA clinical trial programme for rituximab, there were few deaths that occurred in patients in the rituximab groups [12,17]. The number of deaths across autoimmune diseases appears to be relatively high considering the short observation period of the current study, even though the proportion of deaths is no greater than that reported in the Spanish registry; however, the follow-up period in the Spanish registry was considerably longer than that of the current study [60]. In placebo-controlled trials investigating the efficacy and safety of rituximab in autoimmune diseases for one year or more, the proportion of deaths appears low (< 3%) [56-59]. The discrepancy in safety profiles between the real-life, observational studies and randomised, controlled trials may be explained by consideration of the study entry criteria. In real-life studies, the entry criteria are more 'relaxed' than in randomised, controlled trials, which often result in higher-risk patients being included; consequently, outcomes such as mortality rates can be elevated in real-life studies. Overall, no specific pattern emerged from this analysis of GRAID in relation to the type of infection leading to death, prior therapies, age, gender and dosing of rituximab. The deaths predominantly occurred in a short amount of time following rituximab therapy; however, patients had previously received intensive immunosuppressive therapy and had uncontrolled severe disease, both of which would enhance the risks of severe infection and life-threatening complications [62,67-71]. In a study of patients with refractory RA [12], an infection was complicated by an existing heart condition during rituximab therapy, which led to the death of a patient, thus illustrating the difficulty in clearly relating cause of death in the context of severe confounding risk factors. In the recent RITUXIVAS study, a relatively high number of deaths occurred early in the course of therapy (median time to death: 81 days) [8]; however, overall 18% of patients died in the control group (cyclophosphamide), which compared well with 18% in the treatment arm (cyclophosphamide + rituximab). Together with the data of the current study with the highest death rates among patients with ANCA-associated granulmatous vasculitides, the recent trial results of RITUXIVAS indicate the challenges of this disease and suggest that previous and co-therapies as well as comorbidities play a substantial role in this complication. Patients in this and the RAVE study also had an elevated number of malignancies in a short time period [8,9], which was not observed in the current GRAID analysis. In these challenging and often difficult to treat conditions, therapeutic options are limited and physicians are often required to weigh up the benefit:risk ratio of newer therapies off-label.

Response to rituximab in patients with refractory RA has been proven in a number of well-designed clinical studies [11-16]. The current analysis and previous analyses of registry data [60,61] provide an indication that the effectiveness of rituximab may not be limited solely to the treatment of RA. In addition, our data are very much consistent with reported open-label experiences [21,22,25,26,30-33,36-38,40,43,44,72]. However, the interpretation of these data is limited without the inclusion of a control group, and some placebo-controlled studies of rituximab in specific autoimmune diseases failed to provide conclusive evidence as to its benefit [9,56-59]. The current study differs from the lupus studies (LUNAR [58] and EXPLORER [59]) in that data were collected from otherwise refractory patients while both controlled studies included patients who were stable on SOC at study entry and received rituximab plus SOC in the active group compared with SOC in the control group. Further endorsement of the benefit of rituximab in a refractory population has been provided by the RAVE study [9]. In a subgroup of patients with ANCA-associated granulomatous vasculitis who had relapsed with SOC (in particular, cyclophosphamide), rituximab was shown to be superior to SOC (*P *= 0.01), whereas in the overall population that included newly diagnosed disease, there was no significant difference shown between rituximab and the control. Therefore, the bulk of evidence would suggest that treatment of autoimmune diseases, other than RA, with rituximab represents a therapeutic option to consider.

An additional interesting analysis of the GRAID data revealed that there were no significant differences in the distribution patterns for complete, partial and no response across the different autoimmune diseases. However, some patient groups were very small and given the lack of prospective use of validated outcome measures, it cannot be excluded that certain diagnoses have distinct response profiles.

With the continued gathering of evidence from patients with autoimmune diseases treated with rituximab in the real-life setting, supplemented with evidence from well-controlled trials, the picture should become clearer as to which autoimmune diseases and which populations, based on disease stage and previous treatments, are suitable for treatment with rituximab. Although it is very important that data are gathered on patients who might usually be excluded from clinical trials, there are several limitations to studies based on registry data. In general, retrospective, observational studies lack the structure and control groups of a randomised, controlled trial, and the inclusion of heterogeneous patient populations and treatment regimens make comparison with other data difficult. In this particular study, the data collected depended on the interpretation of the individual, participating physicians, and so there may be a lack of consistency in defining certain events and responses. Small patient numbers in any study reduce the power for observing a difference among groups. In the current study, small patient numbers for certain autoimmune diseases may have skewed the results, and subsequent interpretation, which may have precluded the identification of different safety profiles as well as different response profiles compared with the overall population. Finally, incomplete patient records and different follow-up observation periods of individual patients may also add to the limitation in these types of study. In particular, this retrospective analysis is limited by the data that have been recorded and captured within the database; therefore, the reporting of IRRs (as mentioned above), the way response was recorded (that is, at the physicians discretion), incomplete data on comorbidities and the lack of long-term follow-up safety data all limit the interpretation of these data.

## Conclusions

These data in a real-life clinical setting indicate that rituximab is frequently used as an off-label therapy, and suggest that rituximab may be an effective and well-tolerated therapy in a number of treatment-refractory autoimmune diseases, in line with results from the French and Spanish registries of patients with various autoimmune diseases [60,61]. Further randomised, controlled studies in addition to collecting further registry data are warranted to evaluate the use of rituximab fully in these conditions, and to confirm the legitimacy of the results from this current analysis. Registry data, in particular, are extremely valuable since they provide information on patient populations usually excluded from clinical trials. Since certain therapies may provide benefit to otherwise refractory patients, it is an ethical obligation to search and evaluate potential therapeutic options for patients in urgent need.

## Abbreviations

AEs: adverse events; AIHA: autoimmune haemolytic anaemia; AITP: autoimmune thrombocytopenia; ANCA: antineutrophil cytoplasmic antibody; ANOVA: analysis of variance; AS: ankylosing spondylitis; CTCAE: Common Terminology Criteria for Adverse Events; eCRF: electronic case report form; GRAID: German Registry of Autoimmune Diseases; IRRs: infusion-related reactions; ICH: International Conference on Harmonization; MCTD: mixed connective tissue disease; MP: microscopic polyangiitis; NCI: National Cancer Institute; NHL: non-Hodgkin's lymphoma; NMO: neuromyelitis optica; PA: psoriatic arthritis; RA: rheumatoid arthritis; SOC: standard of care; SAEs: serious AEs; SLE: systemic lupus erythematosus; VAS: visual analogue scale; WG: Wegener's granulomatosis.

## Competing interests

The authors declare that they have no competing interests.

## Authors' contributions

All authors of the different disciplines actively enrolled patients into the study. TD, HPT, GRB, HS-K and CS determined the study design. KF, ES and WJ collected the data and KF performed the statistical analysis. TD drafted the manuscript and all authors critically reviewed it for important intellectual content. All authors had access to the data and approved the final version of the manuscript.

## Supplementary Material

Additional file 1 **Supplemental tables**. Table A1. Duration of follow-up from first rituximab infusion to last control visit by diagnosis. Table A2. Number of rituximab infusions by diagnosis. Click here for file
