# Supplementary material for: Safety and clinical outcomes of rituximab therapy in patients with different autoimmune diseases: experience from a national registry (GRAID)
Source: Arthritis Res Ther. 2011 May 13;13(3):R75. doi: 10.1186/ar3337 (PMC3218885; doi:10.1186/ar3337)
Supplement: Additional file 1 — Supplemental tables. Table A1. Duration of follow-up from first rituximab infusion to last control visit by diagnosis. Table A2. Number of rituximab infusions by diagnosis. [file ar3337-S1.DOC]

**Online supplement**

**Table A1. Duration of follow-up from first rituximab infusion to last control visit by diagnosis**

| **Diagnosis** | **Patients, n (%)** | **Median (range) follow-up, months** |
| --- | --- | --- |
| ***Total*** | ***370*** | ***6.5 (0–56.6)*** |
| Arthritis (non-RA)/ ankylosing spondylitis/ psoriatic arthritis | 6 (1.6) | 4.4 (0–9.0) |
| Autoimmune haemolytic anaemia | 3 (0.8) | 8.5 (3.7–30.9) |
| Autoimmune thrombocytopenia | 10 (2.7) | 6.2 (2.0–32.0) |
| Glomerulonephritis | 9 (2.4) | 8.5 (0–24.5) |
| Cryoglobulinaemic vasculitis | 5 (1.4) | 8.3 (0–23.7) |
| Wegener’s granulomatosis/ microscopic polyangiitis | 58 (15.7) | 16.8 (0–56.6) |
| Multiple sclerosis/ neuromyelitis optica | 56 (15.1) | 7.1 (0–29.7) |
| Myasthenia gravis | 5 (1.4) | 5.6 (0–35.1) |
| Pemphigus | 37 (10.0) | 11.6 (1.1–36.9) |
| Sjögren’s syndrome | 6 (1.6) | 6.2 (0–25.1) |
| Polydermatomyositis | 26 (7.0) | 8.5 (0–33.7) |
| Systemic lupus erythematosus | 85 (23.0) | 7.5 (0–49.8) |
| Vasculitis | 13 (3.5) | 3.7 (0–19.2) |
| Overlap syndromes: mixed connective tissue disease | 19 (5.1) | 0 |
| Others^a^ | 32 (8.6) | 0 |

**Table A2. Number of rituximab infusions by diagnosis**

| **Diagnosis** |  | **Number of rituximab infusions** | | | | | | | | | | | | | | | | | |
| --- | --- | --- | --- | --- | --- | --- | --- | --- | --- | --- | --- | --- | --- | --- | --- | --- | --- | --- | --- |
|  |  | **1** | | **2** | | **3** | | **4** | | **5** | | **6** | | **7** | | **8** | | **>8** | |
|  | **N** | **n** | **%** | **n** | **%** | **n** | **%** | **n** | **%** | **n** | **%** | **n** | **%** | **n** | **%** | **n** | **%** | **n** | **%** |
| ***Overall*** | ***370*** | ***50*** | ***13.5*** | ***145*** | ***39.2*** | ***15*** | ***4.1*** | ***126*** | ***34.1*** | ***9*** | ***2.4*** | ***5*** | ***1.4*** | ***3*** | ***0.8*** | ***14*** | ***3.9*** | ***3*** | ***0.8*** |
| Arthritis (non-RA)/ankylosing spondylitis/psoriatic arthritis | 6 | . | . | 5 | 83.3 | . | . | 1 | 16.7 | . | . | . | . | . | . | . | . | . | . |
| Autoimmune haemolytic anaemia | 3 | . | . | 1 | 33.3 | . | . | 2 | 66.7 | . | . | . | . | . | . | . | . | . | . |
| Autoimmune thrombocytopenia | 10 | 1 | 10.0 | 1 | 10.0 | . | . | 7 | 70.0 | 1 | 10.0 | . | . | . | . | . | . | . | . |
| Glomerulonephritis | 9 | 1 | 11.1 | 4 | 44.4 | 1 | 11.1 | 2 | 22.2 | . | . | . | . | . | . | 1 | 11.1 | . | . |
| Cryoglobulinaemic vasculitis | 5 | . | . | 2 | 40.0 | . | . | 2 | 40.0 | 1 | 20.0 | . | . | . | . | . | . | . | . |
| Wegener’s granulomatosis/microscopic polyangiitis | 58 | 3 | 5.2 | 17 | 29.3 | 3 | 5.2 | 23 | 39.7 | 2 | 3.4 | . | . | 1 | 1.7 | 8 | 13.8 | 1 | 1.7 |
| Multiple sclerosis/neuromyelitis optica | 56 | 3 | 5.4 | 15 | 26.8 | 4 | 7.1 | 31 | 55.4 | . | . | . | . | 1 | 1.8 | 1 | 1.8 | 1 | 1.8 |
| Myasthenia gravis | 5 | 1 | 20.0 | 2 | 40.0 | 1 | 20.0 | 1 | 20.0 | . | . | . | . | . | . | . | . | . | . |
| Pemphigus | 37 | 16 | 43.2 | 11 | 29.7 | . | . | 8 | 21.6 | . | . | . | . | 1 | 2.7 | 1 | 2.7 | . | . |
| Sjögren’s syndrome | 6 | 1 | 16.7 | 3 | 50.0 | . | . | 2 | 33.3 | . | . | . | . | . | . | . | . | . | . |
| Polydermatomyositis | 26 | 3 | 11.5 | 14 | 53.8 | . | . | 5 | 19.2 | 1 | 3.8 | 1 | 3.8 | . | . | 1 | 3.8 | 1 | 3.8 |
| Systemic lupus erythematosus | 85 | 10 | 11.8 | 41 | 48.2 | 4 | 4.7 | 24 | 28.2 | 1 | 1.2 | 3 | 3.5 | . | . | 2 | 2.4 | . | . |
| Vasculitis | 13 | 2 | 15.4 | 7 | 53.8 | . | . | 4 | 30.8 | . | . | . | . | . | . | . | . | . | . |
| Overlap syndromes: mixed connective tissue disease | 19 | . | . | 12 | 63.2 | 1 | 5.3 | 3 | 15.8 | 3 | 15.8 | . | . | . | . | . | . | . | . |
| Other | 32 | 9 | 28.1 | 10 | 31.3 | 1 | 3.1 | 11 | 34.4 | . | . | 1 | 3.1 | . | . | . | . | . | . |
